# *In vitro* evaluation of amino acid prodrugs of novel antitumour 2-(4-amino-3-methylphenyl)benzothiazoles

**DOI:** 10.1038/sj.bjc.6600225

**Published:** 2002-04-22

**Authors:** T D Bradshaw, M-S Chua, H L Browne, V Trapani, E A Sausville, M F G Stevens

**Affiliations:** Cancer Research Laboratories, School of Pharmaceutical Sciences, University of Nottingham, Nottingham NG7 2RD, UK; Developmental Therapeutics Program, Division of Cancer Treatment and Diagnosis, National Cancer Institute, NIH, Frederick, Maryland, MD 21702-1201, USA

**Keywords:** 2-(4-aminophenyl)benzothiazole, prodrug, CYP1A1

## Abstract

Novel 2-(4-aminophenyl)benzothiazoles possess highly selective, potent antitumour properties *in vitro* and *in vivo*. They induce and are biotransformed by cytochrome P450 (CYP) 1A1 to putative active as well as inactive metabolites. Metabolic inactivation of the molecule has been thwarted by isosteric replacement of hydrogen with fluorine atoms at positions around the benzothiazole nucleus. The lipophilicity of these compounds presents limitations for drug formulation and bioavailability. To overcome this problem, water soluble prodrugs have been synthesised by conjugation of alanyl- and lysyl-amide hydrochloride salts to the exocyclic primary amine function of 2-(4-aminophenyl)benzothiazoles. The prodrugs retain selectivity with significant *in vitro* growth inhibitory potency against the same sensitive cell lines as their parent amine, but are inactive against cell lines inherently resistant to 2-(4-aminophenyl)benzothiazoles. Alanyl and lysyl prodrugs rapidly and quantitatively revert to their parent amine in sensitive and insensitive cell lines *in vitro*. Liberated parent compounds are sequestered and metabolised by sensitive cells only; similarly, CYP1A1 activity and protein expression are selectively induced in sensitive carcinoma cells. Amino acid prodrugs meet the criteria of aqueous solubility, chemical stability and quantitative reversion to parent molecule, and thus are suitable for *in vivo* preclinical evaluation.

*British Journal of Cancer* (2002) **86**, 1348–1354. DOI: 10.1038/sj/bjc/6600225 www.bjcancer.com

© 2002 Cancer Research UK

## 

Novel 2-(4-aminophenyl)benzothiazoles possess remarkable and intriguing antitumour properties (Shi *et al*, 1996; Bradshaw *et al*, 1998a) representing a mechanistic class distinct from clinically used chemotherapeutic agents. Consistently, they are active against only a specific subset of human cancer cell lines in the National Cancer Institute (NCI) *in vitro* anticancer drug screen, producing mean graph patterns that are highly characteristic of this class of compounds only. Moreover, growth inhibition *in vitro* is characterised by a unique biphasic dose-response relationship (Bradshaw *et al*, 1998a,b). *In vivo*, 2-(4-amino-3-methylphenyl)benzothiazole compound **1** outperformed the 3′-halogeno counterparts in breast, colon and ovarian xenograft studies.

Compound **1** is efficiently sequestered by sensitive cell lines (e.g. breast MCF-7, MDA-468; renal TK-10) (Chua *et al*, 1999; Kashiyama *et al*, 1999). CYP1A1 mRNA (Hose *et al*, 2001; Monks *et al*, 2001) activity and protein expression (Chua *et al*, 2000) are induced exclusively in sensitive cell lines. Covalent binding, detected between compound **1** and recombinant CYP1A1, requires metabolism and is significantly reduced by glutathione (Chua *et al*, 2000). This suggests that an electrophilic, reactive intermediate species is formed. The *C*-6 oxidation biotransformation product, liberated into nutrient media, however, is devoid of antitumour activity (Kashiyama *et al*, 1999). Moreover, this metabolite antagonises cellular uptake of compound **1**, covalent binding between CYP1A1 and compound **1**, CYP1A1 activity and growth inhibition induced by compound **1**. In addition, hydroxy derivatives of compound **1** possess mitogenic properties at μM concentrations (Hutchinson *et al*, 2001). In contrast, insensitive cell lines (e.g. breast MDA-MB-435; renal A498, CAKI-1; prostate PC-3) neither retain nor metabolise compound **1** (Kashiyama *et al*, 1999).

Fluorinated analogues of 2-(4-aminophenyl)benzothiazoles have been synthesised which successfully block *C*-oxidation (Hutchinson *et al*, 2001). 2-(4-Amino-3-methylphenyl)-5-fluorobenzothiazole (compound **2**) is the favoured analogue for clinical consideration possessing enhanced efficacy *in vitro* and superior potency *in vivo* against human breast and ovarian tumour xenografts implanted in nude mice. 2-(4-Aminophenyl)benzothiazoles, which are synthetically accessible, small and lipophilic pose a pharmaceutical challenge, as aqueous i.v. formulations would be desired to minimise the possibility of first pass deactivating metabolism and improve drug bioavailability.

Amino acid conjugation may be utilised to enhance the aqueous solubility of amine or alcohol drugs (as amide or ester prodrugs respectively). The primary aromatic amine, dapsone, has been derivatised as amino acid amides fulfilling the criteria for a suitable prodrug–water solubility, chemical stability and rapid, quantitative bioreversion to the parent moiety (Pochopin *et al*, 1995). The exocyclic primary amine function of 2-(4-amino-3-methylphenyl)benzothiazoles has been successfully conjugated to alanine and lysine residues as the mono- and dihydrochloride salts respectively (Hutchinson *et al*, 2002); such structural manipulation converts lipophilic benzothiazoles into water soluble prodrugs (Figure 1

**Figure 1:**
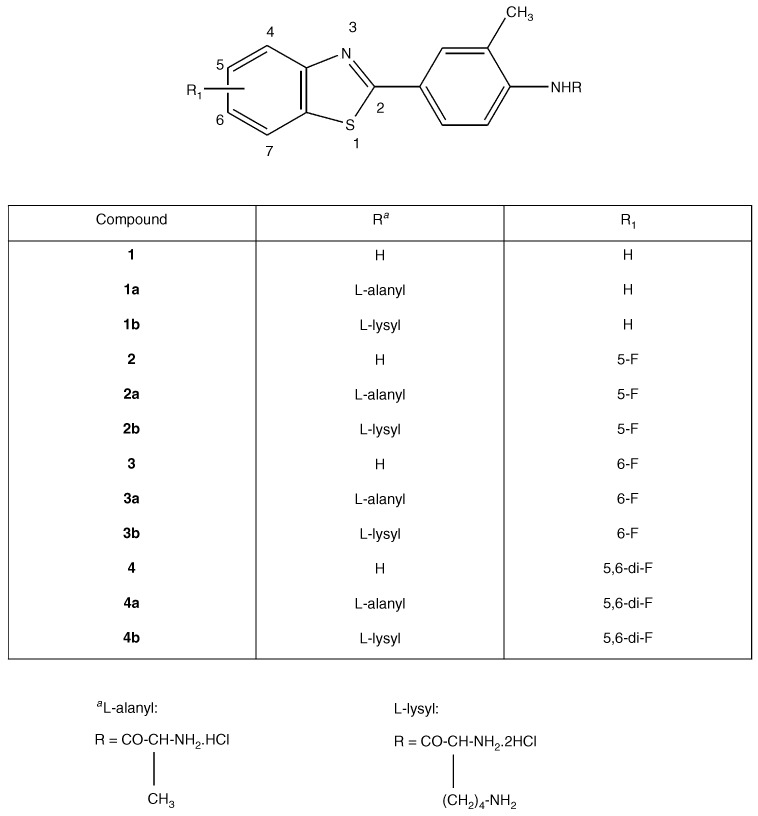
Structures of 2-(4-aminophenyl)benzothiazoles and their amino acid prodrugs.

). Herein, reversion of prodrugs to parent molecule and selective antitumour activity *in vitro* has been described. In addition, we demonstrate that interaction with the putative molecular target of this class of agent, CYP1A1, has not been compromised.

## MATERIALS AND METHODS

### Growth inhibitory assays

Prodrugs were prepared as 10 mM top stocks, dissolved in dimethylsulphoxide (DMSO) or sterile ddH_2_O, and stored at 4°C, protected from light for a maximum period of 4 weeks. MCF-7 (ER+) and MDA 468 (ER−) human derived breast carcinoma cells, cultivated at 37°C in an atmosphere of 5% CO_2_ in RPMI 1640 medium supplemented with 2 mM L-glutamine and 10% foetal calf serum, were routinely subcultured twice weekly to maintain continuous logarithmic growth. Cells were seeded into 96-well microtiter plates at a density of 5×10^3^ per well and allowed 24 h to adhere before drugs were introduced (final concentration 0.1 nM–100 μM, *n*=8). Serial drug dilutions were prepared in medium immediately prior to each assay. Viable cell masses at the time of drug addition (time-zero), and following 72 h drug exposure were determined by cell-mediated 3-(4,5-dimethylthiazol-2-yl)-2,5-diphenyltetrazolium bromide (MTT) reduction. MTT was added to each well (final concentration 400 μg ml^−1^) and plates were incubated at 37°C for 4 h to allow reduction of MTT by viable cell dehydrogenases to an insoluble formazan product. Well supernatants were aspirated and cellular formazan solubilised by addition of DMSO : glycine buffer (pH 10.5) (4 : 1). Cell growth as well as drug activity were determined by measuring absorbance at 550 nm using an Anthos Labtec systems plate reader.

### NCI *in vitro* cytotoxicity assays

Cell culture and drug application procedures have been described previously (Boyd and Paull, 1995). Briefly, cell lines were inoculated into a series of 96-well microtiter plates, with varied seeding densities depending on growth characteristics of each cell line. Following a 24 h drug free incubation, test agents were added at five 10-fold dilutions with a maximum concentration of 100 μM. Cellular protein levels were determined after 48 h drug exposure by sulphorhodamine B colorimetry.

### Metabolism studies

MCF-7 cells were seeded into 25 ml flasks at appropriate densities (5×10^5^–5×10^6^). After 24 h, medium was changed and drug introduced at a final concentration of 10 μM. Media samples, collected from flasks at time zero and 24 h intervals, were mixed with 1.5-fold volumes of high performance liquid chromatography (HPLC) grade acetonitrile to precipitate protein and centrifuged at 14 000 **g** for 5 min. Supernatants (600 μl) were mixed with 400 μl 10% acetonitrile in 1% acetic acid and analysed by HPLC. The system consisted of Beckman System Gold equipment (solvent module 128, autosampler 507e and multiple wavelength UV detector 168). Separation of parent compounds and biotransformation products was effected at room temperature on a Phenomenex Aqua C18 reversed-phase column (150×4.6 mm). The mobile phase was formed by increasing, then decreasing the acetonitrile concentration in 1% acetic acid over 20 min by mixing two solutions: acetonitrile-water-acetic acid (10 : 90 : 1, v v^−1^) and acetonitrile-water-acetic acid (80 : 20 : 1 v v^−1^) and delivered at a flow rate of 1 ml min^−1^. Compounds were detected at 324 nm.

### Western blot protocol

Whole cell lysates were prepared for examination of CYP1A1 protein expression from untreated MCF-7, IGROV-1 and HCT 116 cultures and following exposure of cells to compounds **1**, **2**, **2a**, **2b**, **3**, **3a**, **3b**, **4**, **4a** and **4b**. Following protein determination (*n*=3, Bradford, 1976) and addition of sample buffer, samples were boiled at 95°C for 5 min and solubilised proteins (50 μg) were separated by sodium dodecyl sulphate (SDS) polyacrylamide gel (10%) electrophoresis. Proteins were electroblotted to polyvinylidene difluoride (PVDF) membranes and probed for CYP1A1 protein with polyclonal antiserum specific for human CYP1A1/1A2 (Gentest Corporation). Secondary antibody was conjugated to alkaline phosphatase, and CYP1A1 was detected following brief (<10 min) incubation with bromochloroindolyl phosphate and nitro-blue tetrazolium in alkaline phosphatase buffer. Molecular weight markers and a positive control of recombinant CYP1A1 (Gentest Corporation), included in all blots, confirmed detection of 52 kDa CYP1A1 protein.

### Determination of ethoxyresorufin *O*-deethylase (EROD activity)

A sensitive and rapid fluorometric assay was used to measure EROD activity (Burke *et al*, 1994). Incubation mixtures (total 1 ml) consisted of 100 mM Tris-HCl (pH 7.4), 50 μM MgCl_2_, 100 μM 7-ethoxyresorufin and 100 μl cell homogenate. Homogenates were prepared following treatment of cells for 24 h with compounds 1, **2**, **2a**, **2b**, **3**, **3a**, **3b**, **4**, **4a** and **4b** or vehicle alone and protein content determined (*n*=3). Thus induction of ethoxyresorufin *O*-deethylation by agents under study, catalysed by CYP1A1 activity, could be determined. Alternatively, microsomes expressing recombinant CYP1A1 (0.1 mg ml^−1^) provided the enzyme source for EROD catalysis, in the presence or absence of drug, in order to determine inhibition of CYP1A1 activity by compounds **1**, **2**, **2a**, **2b**, **3**, **3a**, **3b**, **4**, **4a** and **4b**. Incubation mixtures were pre-incubated for 5 min at 37°C before initiation of reaction by addition of NADPH (500 μM). Following further incubations at 37°C (30 min for cell homogenates, 15 min for CYP1A1 microsomes), reactions were terminated by addition of 3 ml ice-cold acetonitrile. Reaction mixtures were centrifuged (1400 r.p.m., 10 min) before analyses of supernatants. Fluorescence was read on a Perkin-Elmer LS-5 luminescence spectrometer (excitation, 530 nm; emission 585 nm). Estimation of resorufin reaction product (pmoles mg^−1^ protein, pmoles 100 μg^−1^ microsomes), as a measure of CYP1A1 activity, was determined following performance of a resorufin standard curve.

## RESULTS

### Prodrug uptake and conversion to parent amine

**Figure 2:**
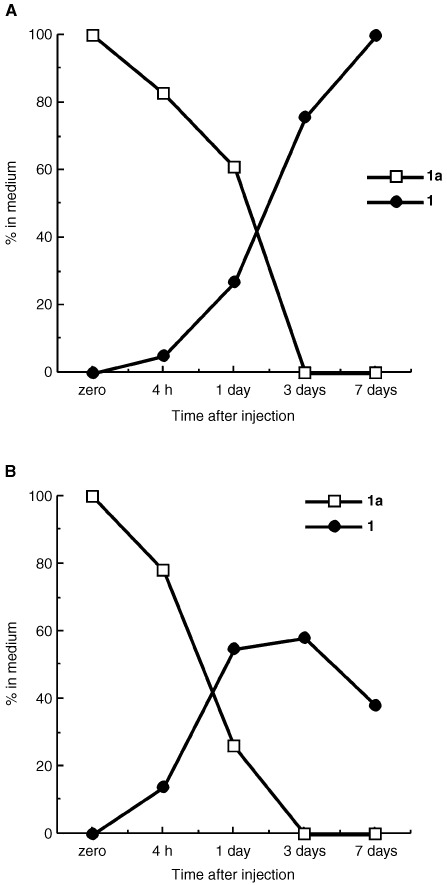
Uptake and conversion of 10 μM compounds **1a** to **1** in: (**A**) inherently insensitive A498 renal cells and (**B**) sensitive ER+ T47D breast cells. Compound **1a** is rapidly and efficiently hydrolysed by both cell lines. Only sensitive cells (**B**) sequester regenerated compound **1**.

Uptake and metabolism of compounds **1a** and **1b** were examined in sensitive breast (MCF-7 and T47D) and insensitive renal (A498) cells using HPLC conditions which allow simultaneous detection of prodrug, parent amine and metabolites. When incubated at 0.1, 1 or 10 μM over 7 days at 37°C, the lysyl-amide compound **1a** was most readily removed from the media supporting MCF-7 and T47D cells (Figure 2

). The decline of each prodrug in media was accompanied by the appearance of compound **1**. Media levels of regenerated compound **1** reached a peak at day 3, thereafter declining as oxidative metabolism by MCF-7 and T47D sensitive cells yielded its inactive 6-hydroxy metabolite. In contrast, negligible net removal of effluxed compound **1** occurred from media supporting the growth of inherently resistant A498 cells, which fail to sequester and metabolise 2-(4-aminophenyl)benzothiazoles (Kashiyama *et al*, 1999). Thus, compound **1**, regenerated from the amide prodrugs accumulated harmlessly in the media of A498 cells over the 7 day incubation period.

**Figure 3:**
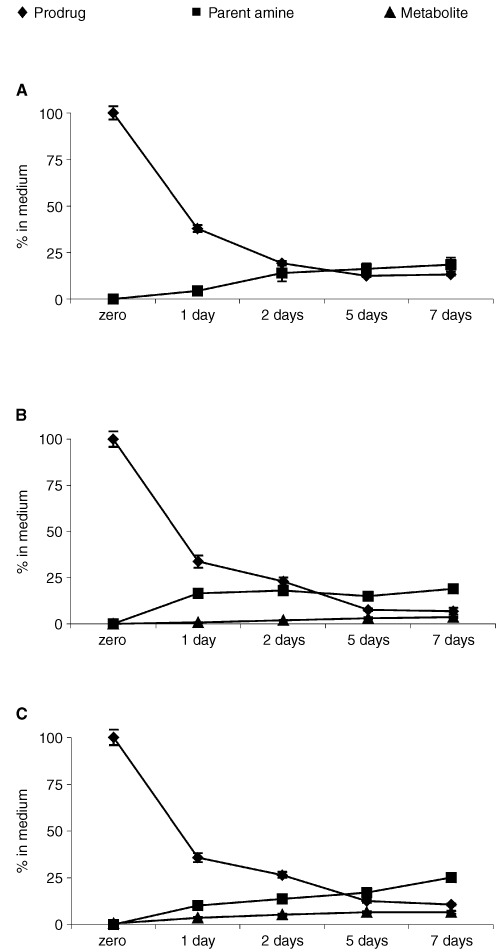
Depletion of (**A**) compound **2a**, (**B**) compound **3a** and (**C**) compound **4a** (10 μM) from nutrient media supporting MCF-7 growth, conversion of prodrugs to the corresponding parent amine and further metabolism of compounds **3** and **4** only by MCF-7 cells (**B** and **C**).

Amino acid prodrugs of compounds **2**, **3** and **4** were stable in medium alone at 37°C>7 days: detection of parent drug during this time was negligible. Uptake and metabolism of compounds **2a**, **2b**, **3a**, **3b**, **4a** and **4b** (10 μM) by MCF-7 cells were examined; conditions for HPLC analysis allowed detection of prodrug, parent compound and metabolites. All alanyl- and lysyl-amide prodrugs were rapidly depleted from nutrient media in the presence of cells and conversion of prodrugs to parent amines was apparent after 24 h. Release of compound **2** into nutrient medium was significant after 24 h and continued to increase throughout the 7 day period. No biotransformation products could be detected (Figure 3A

). In contrast, emergence of an oxidative metabolite accompanied detection of compounds **3** and **4** (days 1–7, Figure 3B,C).

### *In vitro* cytotoxicity

**Figure 4:**
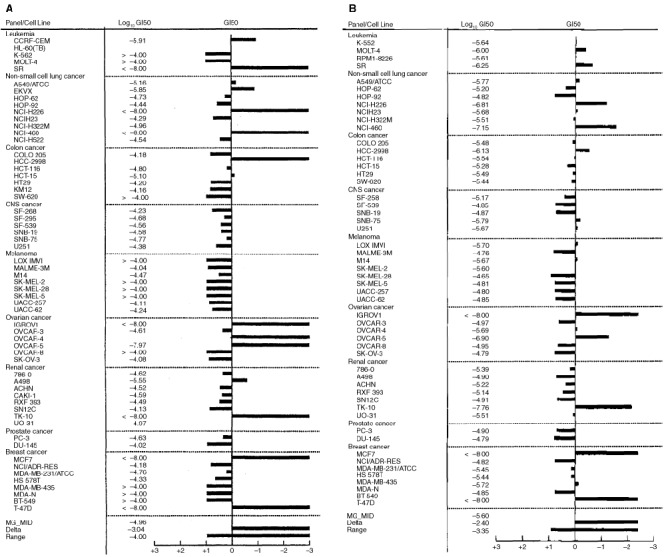
Mean GI_50_ graph demonstrating the selective nature of growth inhibition by (**A**) compound **2** and (**B**) compound **2b** in the NCI *in vitro* panel of human derived cancer cell lines. Cells were exposed to drug for 48 h before viability was determined using the sulforhodamine B assay. Display of data utilises the mean graph format where the average GI_50_ concentration is plotted on the centre line. Using a log scale, the sensitivity or resistance of a particular cell line is represented by deflections to the right or left, respectively, from the mean.

In the NCI panel of human-derived carcinoma cell lines, tested amino acid salts of compounds **1**, **2** and **3** retained the selective growth inhibitory properties of their parent amines; mean graphs demonstrated activity against certain ovarian, renal and breast cell lines following standard 48 h exposure (e.g. Figure 4

). However, as expected considering the prodrug nature of compound **2b**, following 48 h exposure, potency at the GI_50_ level in sensitive cell lines was reduced. Data obtained following examination of activity of amino acid salts of compounds **1**, **2**, **3** and **4** in MCF-7 and MDA 468 breast cancer cells corroborates this observation: typically, after 72 h exposure, GI_50_ values were two orders of magnitude greater than the parent amine (Table 1

**Table 1:**
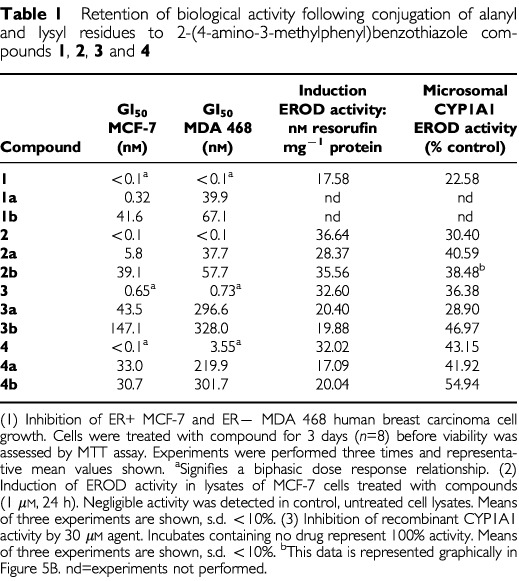
Retention of biological activity following conjugation of alanyl and lysyl residues to 2-(4-amino-3-methylphenyl)benzothiazole compounds **1**, **2**, **3** and **4**

). However, neither alanyl nor lysyl prodrugs evoked the biphasic dose response characteristic of sensitive cell lines exposed to non-conjugated parent amines compounds **1**, **3** or **4**.

### Mechanism of action studies

Figure 5

**Figure 5:**
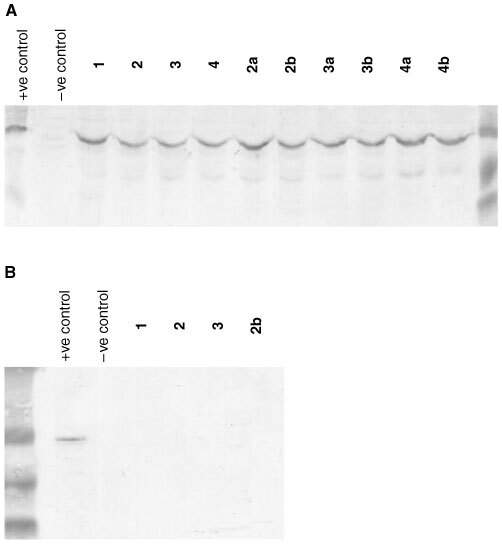
Western Blot detection of CYP1A1 protein induced in lysates of (**A**) MCF-7 cells exposed to compounds **1**, **2**, **3**, **4**, **2a**, **2b**, **3a**, **3b**, **4a** and **4b** (1 μM, 24 h). CYP1A1 expression was not detected constitutively in lysates of untreated MCF-7 or (**B**) HCT 116 cells and not induced in HCT 116 cells exposed to compounds **1**, **2**, **3** or **2b**. For each sample, 50 μg total protein was loaded onto the gel.

demonstrates selective induction of CYP1A1 protein expression in lysates of MCF-7 cells treated with benzothiazole prodrugs and their parent amines (1 μM, 24 h). No CYP1A1 was detected in lysates of HCT 116 or untreated MCF-7 cells. In addition, expression of CYP1A1 protein was clearly observed in lysates of IGROV-1 cells after exposure to concentrations of compound **2b** between 10 nM and 10 μM (24 h, result not shown). Similarly, only lysates of sensitive cells exposed to prodrugs effected deethylation of ethoxyresorufin, indicative of CYP1A1 activity (Table 1). Figure 6A

**Figure 6:**
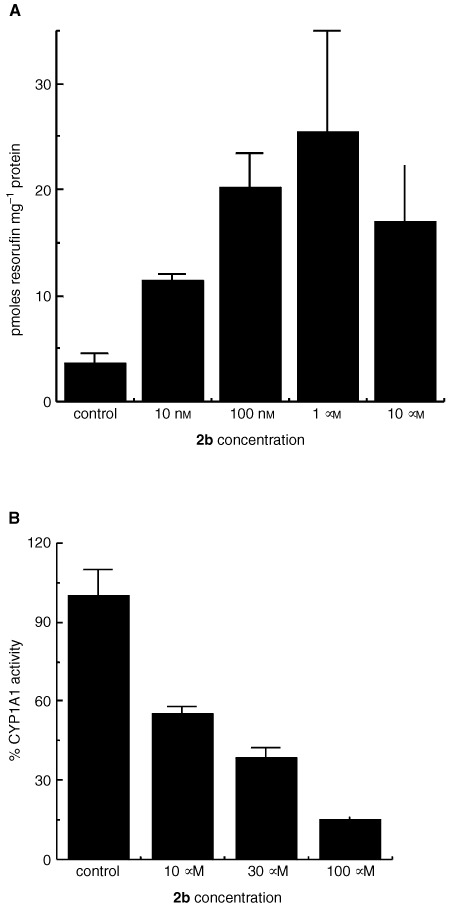
(**A**) EROD activity in homogenates of IGROV-1 cells treated with concentrations of compound **2b** for 24 h. Mean values of three experiments are shown; bars, s.d. (**B**) Inhibition of CYP1A1 microsomal EROD activity by compound **2b**. Resorufin product was measured after coincubation of CYP1A1 microsomes with compount **2b** for 15 min at 37°C. Mean values of three experiments are shown; bars, s.d.

demonstrates the biphasic dose response relationship between CYP1A1-catalysed conversion of ethoxyresorufin to resorufin and compound **2b** concentration in lysates of IGROV-1 cells exposed to this agent for 24 h. Maximum induction of EROD activity followed treatment of cells with 1 μM compound **2b**. It has been speculated that these agents are metabolised by CYP1A1 to a reactive intermediate that covalently binds to, and then inactivates the enzyme; the activity of CYP1A1 microsomes was irreversibly inhibited by compound **1** (Chua *et al*, 2000). Indeed, significant inhibition of CYP1A1 microsomal EROD activity was monitored when incubates included compounds **1**, **2**, **2a**, **2b**, **3**, **3a**, **3b**, **4**, **4a**, **4b** (Table 1, Figure 6B).

## DISCUSSION

Data have been presented describing the *in vitro* biological properties of alanyl and lysyl amide derivatives of 2-(4-amino-3-methylphenyl)benzothiazoles. Prodrugs are rapidly depleted from nutrient media, generating their parent species in the presence of cells (Figures 2 and 3). In the absence of cells, no spontaneous hydrolysis of the amide bond occurs to release the amine from its amino acid salt. The cleavage of amino acid amides may be catalysed by ubiquitously expressed aminopeptidases, but subsequent bioactivating steps, essential for the mechanism of action of aminophenylbenzothiazoles, take place selectively in sensitive cell lines (e.g. Figure 5). Compounds **1a** and **1b** were stable when incubated at 37°C with human plasma; specifically, no free amine was detected. Amino acid amide stability, assessed in aqueous solution over a pH range of 4–9, was found to be pH dependent with more acidic pH favouring greater stability (Hutchinson *et al*, 2002).

The selective depletion of amine (e.g. compound **1**, Figure 2b) from nutrient media *in vitro*, and accumulation in sensitive cells only has been reported previously (Kashiyama *et al*, 1999; Chua *et al*, 2000).

The remarkably potent and selective antitumour activity characteristic of this class of agent is retained; total growth inhibition (TGI) and cytocidal effects (LC_50_) were elicited by nM concentrations following amino acid conjugation coupled with monofluoro substitution. *In vitro*, prodrug compound **2b** exhibits a superior antitumour profile. *In vivo*, plasma concentrations of compound **2**, regenerated from lysylamide prodrug compound **2b**, sufficient to elicit cytocidal activity against human mammary carcinoma cells persist >6 h (Bradshaw *et al*, 2002). Indeed, compound **2b** suppresses significantly the growth of breast and ovarian xenografts *in vivo*.

Planar, hydrophobic 2-(4-aminophenyl)benzothiazoles fulfil structural requirements for binding to the aryl hydrocarbon receptor (AhR), and are potent AhR agonists (Loaiza-Perez *et al*, 2002). Subsequent induction of CYP1A1 mRNA (Hose *et al*, 2001; Monks *et al*, 2001) activity and protein expression (Chua *et al*, 2000; Hutchinson *et al*, 2001) only in cell lines sensitive to the growth inhibitory properties of 2-(4-amino-3-methylphenyl)benzothiazoles has been reported. That the role of CYP1A1 as a putative target for the mechanism of action of this class of compound was not compromised following prodrug modification of the 2-(4-aminophenyl)benzothiazole structure has been verified (Table 1, Figures 5 and 6). Induction of CYP1A1 protein expression and EROD activity in lysates of sensitive MCF-7 breast, IGROV-1 ovarian cells was observed following exposure to prodrugs. Lysates of insensitive HCT 116 cells treated with prodrugs neither expressed CYP1A1 protein nor effected ethoxyresorufin *O*-deethylation.

Induction of CYP1A1 activity and protein expression probably leads to generation of a reactive electrophilic species and benzothiazole-derived covalent binding to CYP1A1, resulting in enzyme inactivation (Chua *et al*, 2000). We have established that at prodrug compound **2b** concentrations >1 μM, EROD activity, induced in lysates of treated sensitive cells, begins to decline (Figure 6A). Moreover, μM prodrug concentrations inhibit microsomal CYP1A1 activity (Figure 6B), substantiating the thesis that these agents are inducers, substrates and suicide inhibitors of CYP1A1. Generation of a CYP1A1-dependent reactive electrophilic species preceeds detection of DNA adducts (Stevens *et al*, 2001), and ultimately cell death.

In conclusion, alanyl- and lysyl-amide prodrugs of 2-(4-amino-3-methylphenyl)benzothiazoles undergo reversion to their parent amine. *In vitro* efficacy and selectivity are retained. Stark antitumour selectivity can be rationalised, and involvement of a crucial molecular target of this class of agent demonstrated. In addition, chemical stability and aqueous solubility is exhibited rendering these synthetically accessible prodrugs suitable for further preclinical development.
